# Marginal and Internal Gap of Metal Copings Fabricated Using Three Types of Resin Patterns with Subtractive and Additive Technology: An In Vitro Comparison

**DOI:** 10.3390/ma15186397

**Published:** 2022-09-15

**Authors:** Hemavardhini Addugala, Vidyashree Nandini Venugopal, Surya Rengasamy, Pradeep Kumar Yadalam, Nassreen H. Albar, Ahmed Alamoudi, Sarah Ahmed Bahammam, Bassam Zidane, Hammam Ahmed Bahammam, Shilpa Bhandi, Deepti Shrivastava, Kumar Chandan Srivastava, Shankargouda Patil

**Affiliations:** 1 Department of Prosthodontics and Implantology, SRM Kattankulathur Dental College and Hospital, SRM Institute of Science and Technology, SRM Nagar, Chennai 603203, India; 2 Independent Researcher, Chennai 603203, India; 3 Department of Periodontics, Saveetha Dental College and Hospitals, Saveetha Institute of Medical and Technical Sciences, Saveetha University, Chennai 602117, India; 4 Department of Restorative Dental Sciences, Division of Operative Dentistry, College of Dentistry, Jazan University, Jazan 45142, Saudi Arabia; 5 Department of Oral Biology, King Abdulaziz University, P.O. Box 80209, Jeddah 21589, Saudi Arabia; 6 Department of Pediatric Dentistry and Orthodontics, College of Dentistry, Taibah University, P.O. Box 344, Medina 42353, Saudi Arabia; 7 Department of Restorative Dentistry, King Abdulaziz University, P.O. Box 80209, Jeddah 21589, Saudi Arabia; 8 Department of Pediatric Dentistry, College of Dentistry, King Abdulaziz University, P.O. Box 80209, Jeddah 21589, Saudi Arabia; 9 Department of Cariology, Saveetha Dental College & Hospitals, Saveetha Institute of Medical and Technical Sciences, Saveetha University, Chennai 600077, India; 10 Department of Preventive Dentistry, College of Dentistry, Jouf University, Sakaka 72388, Saudi Arabia; 11 Department of Oral & Maxillofacial Surgery & Diagnostic Sciences, College of Dentistry, Jouf University, Sakaka 72345, Saudi Arabia; 12 Department of Maxillofacial Surgery and Diagnostic Science, Division of Oral Pathology, College of Dentistry, Jazan University, Jazan 45142, Saudi Arabia; 13 Centre of Molecular Medicine and Diagnostics (COMManD), Saveetha Dental College & Hospitals, Saveetha Institute of Medical and Technical Sciences, Saveetha University, Chennai 600077, India

**Keywords:** computer-aided manufacturing, internal gap, marginal gap, 3D printing, replica technique

## Abstract

This study analyzes the evidence of the marginal discrepancy and internal adaptation of copings fabricated using three types of resin patterns with subtractive (milling) and additive technology (3D printing), as it is not widely reported. Working casts (*n* = 15) were scanned and patterns were completed using computer-aided designing (CAD). Resin patterns were fabricated using the designed data and divided into three groups according to the method of fabrication of patterns: subtractive technology–CAD milled polymethyl methacrylate resin (Group-PMMA), additive technology [digital light processing (DLP) technique]–acrylonitrile–butadiene–styrene (ABS) patterns (Group-ABS), and polylactic acid (PLA) patterns (Group-PLA). Resin patterns were casted with Cobalt–Chromium (Co–Cr) alloy (lost wax technique). Internal and marginal gaps of the metal copings were analyzed with the replica technique under optical microscope. The Kruskal–Wallis test was used to compare values among the groups, and post hoc multiple tests confirmed the specific differences within the groups. The median marginal gap was least for CAD milled resin patterns, followed by PLA printed resin patterns and ABS printed resin patterns. There were significant differences between Group-PMMA and Group-PLA and Group-ABS (*p* = 0.0001). There was no significant difference between Group-PLA and Group-ABS (*p* = 0.899). The median internal gap was least for metal copings fabricated from Group-PLA, followed by Group-ABS and Group-PMMA. The differences were not statistically significant (*p* = 0.638) for the internal gap. Full metal Co–Cr copings fabricated from the milled PMMA group had a better marginal fit, followed by the PLA and ABS printed groups. Copings fabricated with the PLA printed group had the best internal fit, though the values were statistically insignificant between the groups.

## 1. Introduction

The precise seating of restoration is essential in dental restorations to fulfill biological, physical, and esthetic requirements. The occurrence of any marginal discrepancy causes dissolution of cement, micro-leakage, and plaque retention, which in turn leads to the accumulation of bacteria, inflammation, and secondary caries. 

Laboratory and clinical factors impact internal and marginal gaps in restorations to a great extent. Laboratory factors include the incompatibility of dental materials such as die stone, pattern materials, die spacer, casting investments, and casting techniques [1,2]. Clinical factors are tooth preparation geometry, degree of taper, type of finish line, and impression materials used for the restoration in a dental office [3,4]. 

Despite the importance of marginal fidelity, there is no consensus on margin opening or misfit that is considered clinically acceptable. Previous studies have reported a wide range of acceptable marginal gap, from 50 μm to 300 μm [5,6,7,8,9]. Von Fraunhofer and McLean stated 120μm as acceptable for clinical use, and it is the most quoted in original research studies [10]. An acceptable pattern fabrication is an important factor influencing the internal and marginal fit of the restorations [11,12].

The evolution of digital technology has made the possibility of fabricating restorations with subtractive or additive methods. Subtractive manufacturing is the process of constructing three-dimensional objects by successively cutting away material from a solid material block. In additive technology/rapid prototyping, 3D objects are built by adding layer-upon-layer of material. The additive technologies reported in literature are selective laser sintering (SLS), digital light processing (DLP), stereo lithography (SL), polyjet, and so on [13]. The patterns obtained from these techniques can be subjected to casting procedures. The accuracy of resin patterns fabricated using the above-mentioned technologies has not been studied extensively. Utilizing technology at this stage of casting process enables the faster fabrication of patterns that can provide uniform quality in all restorations in lesser time. 

However, there is limited available literature about the fabrication of restorations using resin patterns for the casting of crowns and on the fit of such restorations [14,15]. There is also a lack of studies comparing the discrepancies of the copings made using pattern resins that were fabricated by subtractive and additive technology in peer-reviewed literature. 

The aim of this present study was to compare the marginal and internal gap of metal copings fabricated using three types of resin patterns with subtractive (milling) and additive (3D printing) technology. The secondary objective was to compare the internal and marginal gaps of the different walls of the preparation.

The study began with the following null hypothesis: there will be no difference in the marginal gap and internal gap of copings fabricated using subtractive and additive technology. 

## 2. Materials and Methods

**Figure 1:**
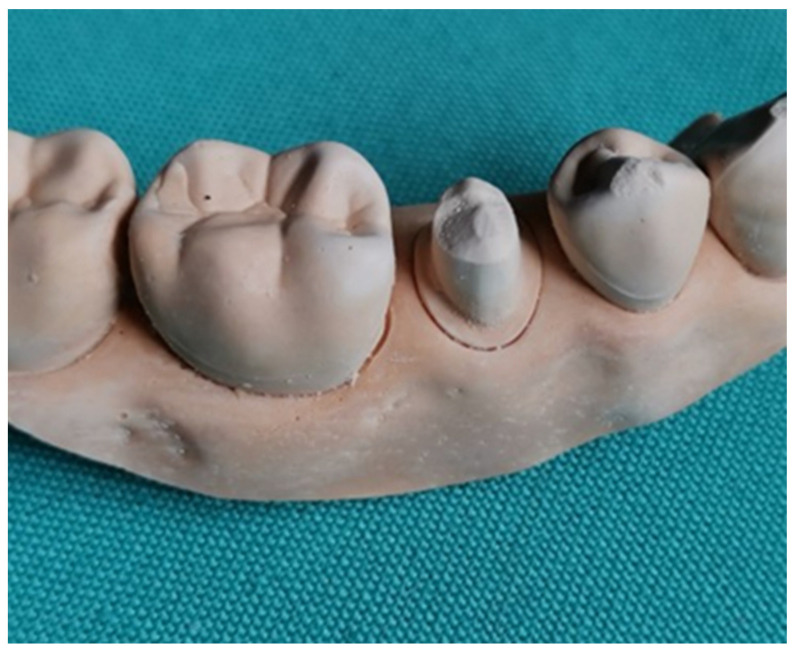
Working cast.

The study was approved by the Institutional Ethical Committee (Ref No.1475/IEC/2018). A Mandibular typodont was utilized as the master model (Model type: D91SHD-200, Nissin Dental products INC. Kyoto, Japan). Tooth preparation was done for a full-coverage restoration in the right second premolar region (45) following the preparation guidelines with 16^0^ total occlusal convergence (TOC) [16]. Fifteen conventional impressions were made using low viscosity and putty consistency polyvinyl siloxane material (Photosil, DPI, Mumbai, India), using the double mix putty–wash technique. Each of the fifteen impressions was sprayed with a debubblizer (Unicoat, Delta, Chennai, India) and poured with Type IV Gypsum (Die stone–Ultrarock, Kalabhai Karson, Mumbai, India) using a vibrator (AX-2000, Aixin Medical Equipment Co., Ltd., Tianjin, China) to make fifteen working casts (Figure 1). 

**Figure 2:**
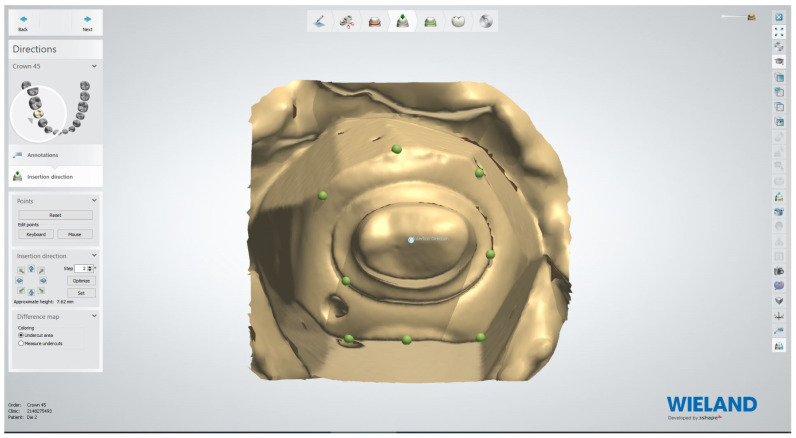
Designing in CAD software.

The working casts were then scanned and digitized using a model scanner (Blue light LED scanner, D900 L, 3 Shape, Copenhagen, Denmark). 3D images of casts were projected on the monitor for designing the resin patterns in the CAD software (3 Shape Dental Manager Dental System 2020 -1 88.1.9 (DS 20.1.2), Copenhagen, Denmark). Designing involves the following steps: i. determining the area of interest, ii. assessing the path of insertion with minimal undercuts, iii. outlining the margins of the patterns, and iv. designing the anatomical contour of the resin patterns (Figure 2). 

The design was saved in STL (standard tessellation language, or standard triangulation language) file format and transferred to the milling software (subtractive technology/CAD/CAM milling method), as well as to the slicing software (additive technology/3D printing additive technology), to get three types of resin patterns such as CAD milled PMMA resin patterns (Group-PMMA, *n* = 15), DLP printed acrylonitrile–butadiene–styrene (ABS) resin patterns (Group-ABS, *n* = 15), DLP printed polylactic acid (PLA) resin pattern (Group-PLA, *n* = 15) (Table 1).

### 2.1. Fabrication of Group-PMMA Samples

**Figure 3:**
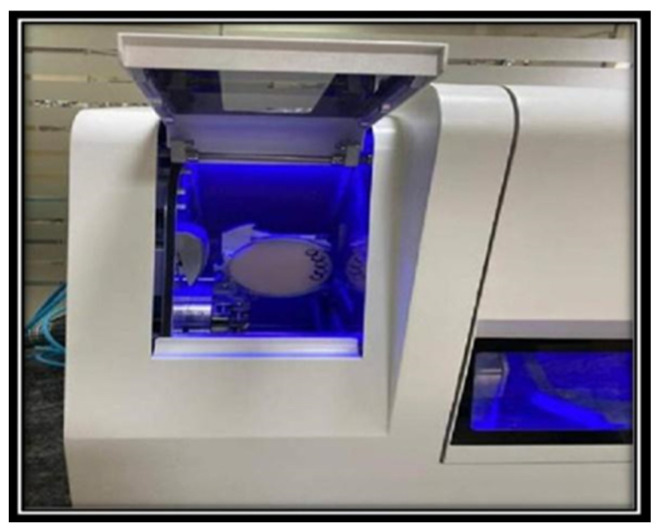
Milling of PMMA resin pattern.

In Group-PMMA, the casts were scanned using the model scanner (Blue light LED scanner, D900 L, 3 Shape, Denmark). Designing of the scanned working cast was done using CAD software (3 Shape Dental System, Denmark). This was followed by design transfer to the milling software as STL files, and the patterns were milled in a CAD–CAM milling unit (Zenotec Hybrid Select, Wieland, Stuttgart, Germany) using a PMMA disc (Figure 3).

A total of 15 resin patterns were milled and placed over their corresponding dies. Wax sprues of 3 mm length were used to join resin patterns and were invested using a phosphate-bonded investment material (Bellavest SH +Begoso, Bego, Bremen, Germany). The casting was done with Co–Cr alloy (Wirobond LFC, Bego, Bremen, Germany) using an induction casting machine (Fornax T-Bego, Bremen, Germany). The castings were sectioned from the sprues by an ultra-fine carborundum disc, and then the metal copings were finished and placed over their respective dies.

### 2.2. Fabrication of Group-ABS (Acrylonitrile–Butadiene–Styrene) Samples

**Figure 4:**
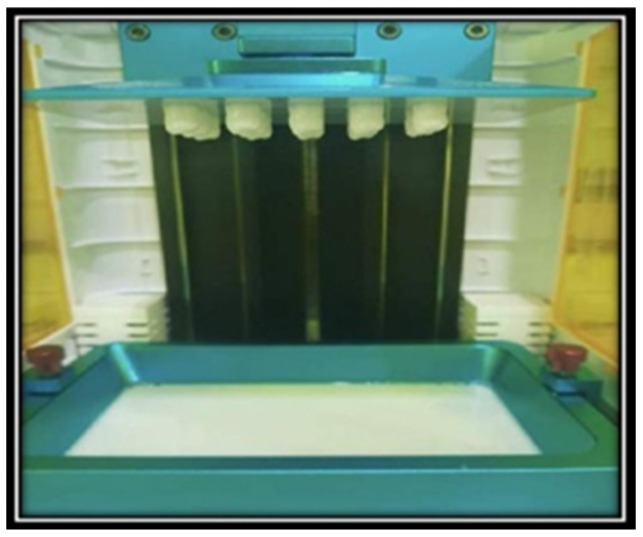
Printing of acrylonitrile–butadiene–styrene (ABS) resin patterns.

The working casts were scanned and digitized similarly to that of Group-PMMA. The STL file was exported to a slicing software (Chitubox v1.6.4.3 Beta) to edit the layers, tooth path, temperature, color, and print speed. The software helped in slicing the model file into layers and generated a specific g-code for the 3D printer. The physical model was printed using this g-code. The 3D printer (Anycubic 3D Printer (Model no. Anycubic Photon S UV, Shenzhen, China)) works by digital light processing (DLP) technique, wherein a light source is projected to cure the photosensitive liquid resin layer-by-layer, following a specific path of the designed model. The building up of the object was done incrementally upside down on an elevating platform, where the occlusal surface was facing downwards towards the resin tank, and the cervical portion was attached to the building platform with the help of supporters. The 120° direction was defined as the orientation angle after positioning the lingual surface of the crown parallel to the build platform and rotating it 30° on the *Y*-axis. The crown was rotated 15° or 30° in the direction of the *Y*-axis until the support was placed on the buccal surface. The support was to be automatically positioned only on the surface that formed an angle of ≥30° with the *Z*-axis. The photopolymer used here had monomers of acrylonitrile–butadiene–styrene (ABS) to fabricate ABS resin patterns. A total of 15 resin patterns were printed (Figure 4). 

The patterns were submerged for 3 min and swirled around in isopropyl alcohol to remove excess resin along with the uncured layer, and also to reduce the residual stickiness. All these resin patterns were cast similarly to that of Group-PMMA to make 15 Co–Cr copings using the lost wax technique.

### 2.3. Fabrication of Group-PLA (Polylactic Acid) Samples

Fabrication of this type of resin pattern was similar to that of Group-ABS. Once the STL files of the fifteen working casts were transferred to a 3D printer, for this group photopolymer, polylactic acid (PLA) resin material was used to print the resin patterns. The printer and printing technique used was the same as for Group-ABS. Only the resin material used was different. All the resin patterns were subjected to the lost wax technique to fabricate 15 Co–Cr copings.

### 2.4. Measurement of Marginal and Internal Gaps

**Figure 5:**
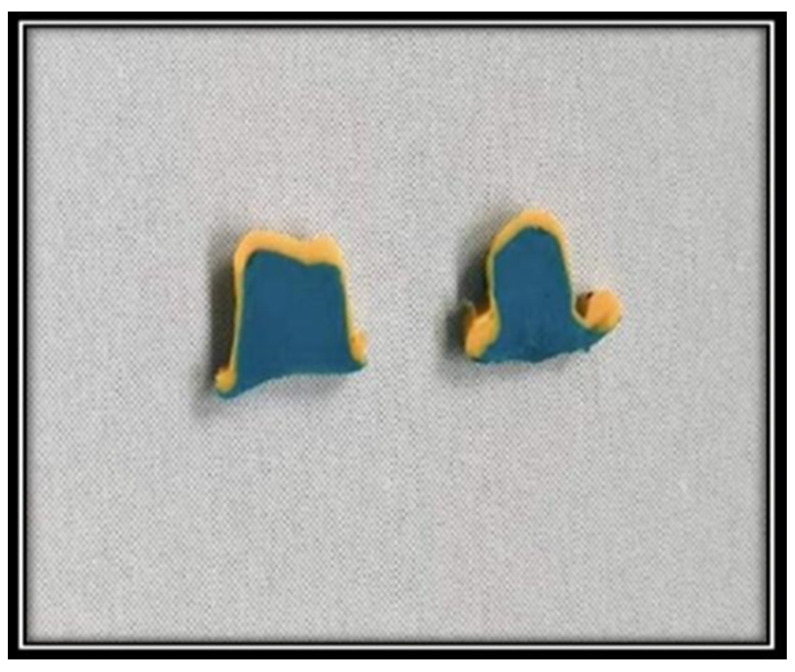
Buccolingual and Mesiodistal sectioned replica.

After the fabrication of crowns, marginal and internal gaps were analyzed by using the replica technique. Marginal and internal gaps were determined according to the terminology previously reported by Holmes [17]. The internal gap is the measurement of the internal surface of the restoration to the axial wall of the preparation in the perpendicular direction. Measurement between the axial wall and internal surface at the margin is called the marginal gap. These gaps are analyzed using the replica technique described by Boening [18]. Using this technique, low viscosity polyvinyl siloxane (Reprosil, Dentsply, Sirona, India) was injected into each coping and placed on the corresponding cast with constant finger pressure for 10 s on the occlusal surface. Once the impression material was set, it was removed from the cast along with the coping. The thin silicon film was supported by another low-viscosity polyvinyl siloxane material (Aquasil LV, Dentsply, Konstanz, Germany) with contrasting color to merge into a single piece with the film [19]. Once the supporting low-viscosity PVS impression material hardened, each replica was removed and sectioned with sharp surgical Bard-Parker blade no.15 buccolingual and mesiodistally [18] (Figure 5). All the procedures were performed by a single operator.

**Figure 6:**
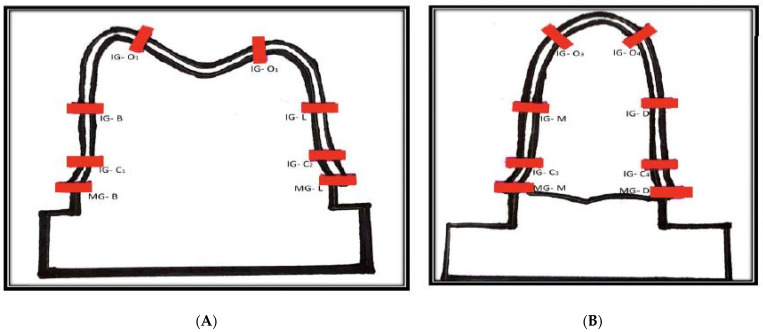
(**A**,**B**): Line diagram showing the location of areas of measurements of the gap on (**A**). Buccolingual section, (**B**) Mesiodistal section.

For each coping, the measurements were obtained from the buccolingual and mesiodistal sections in sixteen locations (eight in the buccolingual section and eight in the mesiodistal section): four marginal (MG-B, MG-L, MG-M, MG-D), four cervical (IG-C1, IG-C2, IG-C3, IG-C4), four axial (IG-B, IG-L, IG-M, IG-D), and four occlusal areas (IG-O1, IG-O2, IG-O3, IG-O4). The locations measured in the buccolingual section are: MG-B, IG-C1, IG-B, IG-O1, IG-O2, IG-L, IG-C2, MG-L (Figure 6A); and in mesiodistal sections are: MG-M, IG-C3, IG-M, IG-O3, IG-O4, IG-D, IG-C4, MG-D (Figure 6B). 

All these measurements were recorded after stabilizing the cross-section of light body impression material with clay over the slide, held under an optical microscope (Leica DMC 2900, Leica Microsystems, Maharashtra, India). The thicknesses were measured using a software tool. 

### 2.5. Statistical Analysis

The data obtained were subjected to normality tests, i.e., Kolmogorov–Smirnov and Shapiro–Wilk tests, which revealed that the distribution of data was non-normal. Hence, the statistical analyses performed were non-parametric tests. The Kruskal–Wallis test was conducted to compare the median values between the three groups. Post hoc Dunn’s multiple comparison test was conducted to determine the significance between the groups.

## 3. Results

The results showed that the median marginal gap was least on the lingual wall in all the groups (PMMA milled group, 48.93 ± 14.19 μm; ABS printed group, 104.48 ± 19.58 μm; PLA printed group, 101.32 ± 19.80 μm). The median marginal gap was the least on all four walls in the resin pattern fabricated from PMMA milled group (48.93 ± 14.19 μm). The median marginal gap was highest on the buccal wall in the resin pattern fabricated from the ABS printed group (111.85 ± 23.83 μm). The median values of PMMA milled resin patterns, ABS printed resin patterns, and PLA printed resin patterns groups were within 120 μm, which happens to be the range of clinical acceptance level. The Kruskal–Wallis test was conducted to find the significance of the marginal gap values obtained using the three different resin patterns. The *p*-value was found to be 0.0001, which is significant as *p* < 0.05 (Table 2). 

The post hoc analysis was done using Dunn’s multiple comparison test between three different resin patterns. Results showed that the comparison of marginal gap values between PMMA milled resin patterns and PLA printed resin patterns, and PMMA milled resin patterns and ABS printed resin patterns, were statistically significant, showing a *p*-value of 0.0001. Conversely, comparison between ABS printed resin patterns and PLA printed resin patterns showed that the difference was not statistically significant (*p* > 0.05) (Table 3). Therefore, from the analysis, we can infer that PMMA milled resin patterns have a marginal gap, followed by PLA printed resin patterns and ABS printed resin patterns.

A comparison of the median internal gap values in the buccolingual cross-section in four different locations revealed that the ABS printed resin patterns group had the highest median internal gap on the occlusal surface (118.31 ± 7.44 µm). the least internal gap was seen in the PLA resin pattern group (107.84 ± 9.43 µm). The median values of PMMA milled resin patterns, ABS printed resin patterns, and PLA printed resin patterns groups were within the range of clinical acceptance (120 µm). However, Kruskal–Wallis analysis showed no statistical significance (*p* > 0.05) (Table 4).

The median internal gap values in the mesiodistal cross-section in four different locations revealed that the PMMA resin pattern group had the highest median internal gap (119.78 ± 8.66 µm). The median values of all the groups were within the range of clinical acceptance (120 µm). Further Kruskal–Wallis analysis showed no statistical significance (*p* > 0.05) (Table 5).

## 4. Discussion

The lost wax technique advocated by Taggart is the widely used method for the fabrication of indirect cast restorations [20]. Traditional casting techniques involve the fabrication of patterns. In this casting technique, wax is usually utilized as the pattern-forming material. One of the disadvantages of the conventional wax pattern is its dimensional inaccuracy. To overcome this drawback, resin pattern material has been introduced. The advantages of the resin materials are rigidity, adequate strength, low polymerization shrinkage, ease of use, lesser chair time, good dimensional stability, and no residue on burnout. An acceptable pattern fabrication is an important factor that influences the internal and marginal fit of the restoration. Fabrication of patterns with wax/resin leads to varied results, as the skill of the person at work determines the outcome. With the increasing influence of digital technology, we have additive and subtractive technologies available to us at affordable costs. The present study compared the marginal gap and internal gap of metal copings fabricated using three different types of pattern resin materials using two different technologies, namely subtractive (milling) and additive (3D printing) technology.

There are several methods for analyzing the internal and marginal gap, such as the cross-sectional method (CSM), silicone replica technique (SRT), triple scan method (TSM), micro-computed tomography (MCT), and optical coherence tomography (OCT). The replica technique is used in many studies since it is a simple, precise, and non-destructive method. In the present study, the replica technique was used to determine the marginal gap and internal gap. This technique has an advantage over other techniques, as it doesn’t require the sectioning of the crown. Possible negative changes might be due to the shrinkage of polyvinyl siloxane, but this technique has been found to be reliable and used in internal gap measurement. Each replica was carefully segmented buccolingually and mesiodistally. Mesiodistal and buccolingual cross-sections were analyzed at sixteen points (eight in the buccolingual section and eight in the mesiodistal section). The replica was measured under an optical microscope at 5× magnification. The thicknesses were measured using a software tool. The results showed that there were significant differences between the three different resin patterns (*p* < 0.05). Hence, post hoc multiple tests were carried out to find if the difference between the groups were significant. Post hoc multiple tests revealed that the marginal gap of the PMMA milled patterns group had statistical significance when compared to ABS and PLA printed patterns groups (*p* = 0.0001). This could be because, in additive processing, parameters that might have the greatest effect on the results are the axes (*z*-axis) of build directions, where the *z*-axis movement is responsible for moving layers at a pre-defined height set in the 3D slicer [21]. The stair-stepping effects may have affected the dimensional accuracy of the pattern.

In the additive technique, each slice or layer is placed on top of the preceding one, resulting in the creation of the model. The thickness of the slices used to manufacture the model can bring about an effect called stepping error, layering error, or staircase effect. The extent of this staircase effect depends on the layer thickness [22]. The staircase error increases with the layer thickness. Distortion of pattern parts can occur during the polymerizing process in additive technique, as it is combined with a lot of heat, thus influencing the interlayer binding. There is a possibility of damage while removing the supporting material after the print is completed. This can increase the marginal gap. Similar results were obtained in the study conducted by Kim et al., and they found that the least marginal and internal gap was seen in crowns fabricated by milling when compared to crowns fabricated using selective laser melting [23]. None of the earlier studies compared the three different resin patterns fabricated using additive and subtractive technology that was cast using the lost wax process; in this way, the current study provides new insights into the available digital technology.

The results of the measured internal gaps showed that the PLA printed resin patterns group had a better internal fit (least internal gap), followed by the ABS printed resin patterns group and the PMMA milled resin patterns group, though the differences were not statistically significant (*p* = 0.638). The mean internal gap obtained from PMMA milled resin patterns, ABS printed resin patterns, and PLA printed resin patterns groups were within the clinically acceptable level of 120 µm. One of the reasons could have been the wear and tear of the milling burs. This can be compensated if the burs are changed often to reduce their decreasing diameter. Milling device vibrations can also be another reason. Similar results were obtained in the study conducted by Elfar M et al. [24]. For each coping, the data were obtained from measurements on sixteen locations in the buccolingual and mesiodistal sections: four marginal, four cervical, four axial, and four occlusal measurements. Though the values were not statistically significant, among all the measured locations, in both techniques, occlusal surfaces showed higher discrepancies, followed by the cervical, marginal, and axial surfaces. This is because the error-prone areas are the curved surfaces, compared to the vertical surfaces affecting the accuracy of the crowns [25]. The occlusal surfaces with larger grooves and fossa make the morphology more complex to print without discrepancies. 

The limitations of the present in vitro study include errors during impression making. Procedures of scanning and software alignment in crown fabrication can also influence results. In the present study, marginal and internal gaps were evaluated using a two-dimensional method. The same can also be studied using the three-dimensional method. The replica technique has the disadvantage of the tearing of the silicone layer replica and errors that occur while sectioning in different planes. For making the replica, the copings were filled with low-viscosity polyvinyl siloxane and seated on the master die using finger pressure. Though this method simulates the clinical situation, the use of finger pressure can result in variability. This study assessed only the copings; future studies can be done with full-contour crowns. Further studies with a long-span fixed dental prosthesis can also be taken up. Clinical trials can validate the study results further.

## 5. Conclusions

The marginal fit was found to be better in milled resin patterns (subtractive technology) than in 3D printed resin patterns (additive technology). The mean marginal gap was found to be maximum in ABS printed resin patterns, followed by PLA printed resin patterns and then in PMMA milled resin patterns. PLA printed resin patterns had a better internal fit, followed by ABS printed resin patterns and then milled resin patterns. The mean marginal and internal gap of metal copings obtained from 3D printed resin patterns (additive technology) and milled PMMA resin patterns (subtractive technology) were within the clinically acceptable range. Digital technologies, both subtractive and additive techniques, showed an increased gap (least fit) on the occlusal surface when compared to the marginal, cervical, and axial surfaces.

## Figures and Tables

**Table 1:** Materials and techniques used for pattern fabrication.

Resin Fabrication Technology	Material–Brand Name, Model and Place of Manufacture	Materials–Composition
Milling	Aidite, 0D9, Hebei, China	Polymethyl methacrylate (PMMA) resin disc 14 mm–A2 shade
3D Printing-Digital Light Processing (DLP)	Weistek, ABS-1000-BL, China	Acrylo-nitrile butadiene styrene (ABS) resin material
3D Printing-Digital Light Processing (DLP)	e-sun, e-resin, PLAgray05A, China	Polylactic acid (PLA) resin material

**Table 2:** Comparison of the marginal gap on all four walls among the three different resin patterns in micrometers.

Resin Pattern Groups (*n* = 15)	Buccal (MG-B)	Lingual (MG-L)	Mesial (MG-M)	Distal (MG-D)	*p*-Value
**PMMA milled**	51.18 ± 8.03	48.93 ± 14.19	69.24 ± 14.16	51.18 ± 8.38	0.307
**ABS printed**	111.85 ± 23.83	104.48 ± 19.58	107.89 ± 20.29	110.08 ± 18.76	0.823
**PLA printed**	108.75 ± 21.90	101.32 ± 19.80	106.89 ± 20.49	109.17 ± 18.94	0.642
***p*-value**	0.0001 ^¶^	0.0001 ^¶^	0.0001 ^¶^	0.0001 ^¶^	

Note: Results are expressed in median ± standard deviation; MG-B—marginal gap on buccal side; MG-L—marginal gap on lingual side; MG-M—marginal gap on mesial side; MG-D—marginal gap on distal side; PMMA—polymethyl methacrylate resin; ABS—acrylonitrile–butadiene–styrene; PLA—polylactic acid; ^¶^ *p* < 0.001.

**Table 3:** Post-hoc Dunn’s multiple comparison test to compare the marginal gap on the four different walls within the groups.

Groups	Walls	Difference in Rank Sum	*p*-Value
PMMA& PLA	Buccal	−66.87	<0.0001 ^¶^
	Lingual	−85.4	<0.0001 ^¶^
	Mesial	−70.07	<0.05 ^¶^
	Distal	−95.67	<0.0001 ^¶^
PMMA & ABS	Buccal	−97.07	<0.0001 ^¶^
	Distal	−107.3	<0.0001 ^¶^
	Lingual	−97.13	<0.0001 ^¶^
	Mesial	−69.27	<0.0001 ^¶^
ABS & PLA	Buccal	30.2	ns
	Lingual	11.73	ns
	Mesial	−0.8	ns
	Distal	11.67	ns

Note: PMMA—polymethyl methacrylate resin; ABS—acrylonitrile–butadiene–styrene; PLA—polylactic acid; ^¶^ *p* < 0.001, statistical significance; ns—no significance (*p* > 0.05).

**Table 4:** Comparison of the internal gap on all six locations among the three different resin patterns in buccolingual cross-section in micrometers.

Resin Pattern Groups (*n* = 15)	Cervical (1) (IG-C1)	Buccal (IG-B)	Occlusal (1) (IG-O1)	Occlusal (2) (IG-O2)	Lingual (IG-L)	Cervical (2) (IG-C2)	*p*-Value
PMMA milled	112.52 ± 12.11	108.39 ± 10.99	117.59 ± 9.27	119.44 ± 9.16	111.11 ± 11.77	116.09 ± 9.04	0.732
ABS printed	108.71 ± 9.77	108.00 ± 9.01	116.50 ± 7.95	118.31 ± 7.44	110.64 ± 7.29	115.42 ± 8.15	0.428
PLA printed	108.10 ± 8.17	106.81 ± 8.67	115.36 ± 7.01	113.81 ± 6.48	107.84 ± 9.43	113.29 ± 9.45	0.503
*p*-value	0.528	0.896	0.687	0.108	0.638	0.753	

Note: Results are expressed in median ± standard deviation; IG-C1—internal gap on cervical side 1; IG-B—internal gap on buccal side; IG-O1—internal gap on occlusal side 1; IG-O2—internal gap on occlusal side 2; IG-L—internal gap on lingual side; IG-C2—internal gap on cervical side 2; PMMA—polymethyl methacrylate resin; ABS—acrylonitrile–butadiene–styrene; PLA—polylactic acid.

**Table 5:** Comparison of the internal gap on all six locations among the three different resin patterns in mesiodistal cross-section using Kruskal–Wallis test.

Resin Pattern Groups (*n* = 15)	Cervical (3) (IG-C3)	Mesial (IG-M)	Occlusal (3) (IG-O1)	Occlusal (4) (IG-O2)	Distal (IG-D)	Cervical (4) (IG-C4)	*p*-Value
PMMA milled	113.50 ± 11.93	111.05 ± 13.59	119.78 ± 8.66	118.81 ± 10.28	112.43 ± 8.86	117.24 ± 11.17	0.427
ABS printed	111.21 ± 7.41	108.37 ± 10.51	116.75 ± 7.33	116.52 ± 7.55	111.87 ± 5.08	115.72 ± 7.00	0.322
PLA printed	110.41 ± 10.05	107.05 ± 10.71	115.89 ± 7.33	116.19 ± 6.96	110.16 ± 8.79	114.57 ± 7.58	0.765
*p*-value	0.673	0.649	0.360	0.654	0.694	0.639	

Note: Results are expressed in median ± standard deviation; IG-C3—internal gap on cervical side 3; IG-M—internal gap on mesial side; IG-O1—internal gap on occlusal side 1; IG-O2—internal gap on occlusal side 2; IG-D—internal gap on distal side; IG-C4—internal gap on cervical side 4; PMMA—polymethyl methacrylate resin; ABS—acrylonitrile–butadiene–styrene; PLA—polylactic acid.

## Data Availability

Not applicable.
